# CD20-Mimotope Peptides: A Model to Define the Molecular Basis of Epitope Spreading

**DOI:** 10.3390/ijms20081920

**Published:** 2019-04-18

**Authors:** Elvira Favoino, Marcella Prete, Giacomo Catacchio, Giuseppina Conteduca, Federico Perosa

**Affiliations:** Department of Biomedical Sciences and Human Oncology (DIMO), Rheumatologic and Systemic Autoimmune Diseases Unit, University of Bari Medical School, I-70124 Bari, Italy; elvira.favoino@uniba.it (E.F.); marcella.prete@uniba.it (M.P.); giaco.catacchio@gmail.com (G.C.); giusy_conteduca@alice.it (G.C.)

**Keywords:** mimotope, peptide, active immunotherapy, antigenicity, immunogenicity, epitope spreading, vaccine, CD20, Rituximab

## Abstract

Antigen-mimicking peptide (mimotope)-based vaccines are one of the most promising forms of active-immunotherapy. The main drawback of this approach is that it induces antibodies that react poorly with the nominal antigen. The aim of this study was to investigate the molecular basis underlying the weak antibody response induced against the naïve protein after peptide vaccination. For this purpose, we analyzed the fine specificity of monoclonal antibodies (mAb) elicited with a 13-mer linear peptide, complementary to theantigen-combining site of the anti-CD20 mAb, Rituximab, in BALB/c mice. Anti-peptide mAb competed with Rituximab for peptide binding. Even so, they recognized a different antigenic motif from the one recognized by Rituximab. This explains their lack of reactivity with membrane (naïve) CD20. These data indicate that even on a short peptide the immunogenic and antigenic motifs may be different. These findings highlight an additional mechanism for epitope spreading and should be taken into account when designing peptides for vaccine purposes.

## 1. Introduction

Targeted immunotherapy has shown considerable clinical efficacy in the treatment of cancer, autoimmune, and infectious diseases [1]. Immunotherapy is classified as either passive or active. Passive immunotherapy involves the administration of immune system components, such as antibodies, to fight the disease, whereas active immunotherapy stimulates the host immune system to generate a durable response against the target antigen by inducing immunological memory. Among the strategies for active immunotherapy developed so far, anti-idiotypic (anti-Id) antibodies vaccines have been widely applied in clinical trials [2,3,4,5,6,7]. However, despite the safety, tolerability, and immunogenicity of anti-Id vaccines, their clinical benefit remains unproven. By contrast, peptide-based vaccines display unique advantages, such as an immune response focused only on relevant epitopes, low cost, and minimal side effects. Even so, no peptide-based vaccine has yet been approved by the Food and Drug Administration (FDA) although many are in different stages of clinical testing [8,9,10,11].

In the CD20 antigen system, the chimeric monoclonal antibody (mAb) Rituximab is a successful example of passive immunotherapy. Indeed, Rituximab has been approved by the FDA for the treatment of non-Hodgkin’s lymphoma, chronic lymphocytic leukemia, and rheumatoid arthritis, and has been proven to be successful in non-controlled clinical studies in treating lupus nephritis, Wegener’s granulomatosis, microscopic polyangiitis, and pemphigus vulgaris, reviewed in [12,13]. In the context of active immunotherapy, Roberts et al. showed that a 40-mer peptide representing the whole extracellular domain of human CD20 was not so effective as a vaccine, because mice developed sera antibodies that reacted poorly with cell surface CD20, despite the high levels of antibodies specifically recognizing the immunogen peptide [14]. In addition, unlike Rituximab, the poorly reacting CD20-specific antibodies also cross-reacted with the mouse counterpart of CD20. This was probably due to the abnormal length of the peptide used as an immunogen, which likely took on a different three-dimensional conformation from that of the nominal antigen, thereby unmasking or expressing novel epitopes.

A better understanding of the reasons why antibodies raised to peptides may react poorly or not at all with the native protein may help to design effective peptide-based vaccines.

On the basis of the above observations, we tested the hypothesis that vaccination with a shorter synthetic peptide bearing the epitope recognized by Rituximab on CD20 could induce a more epitope-focused, -effective immune response.

The panning of phage display peptide libraries (PDPLs) with Rituximab led to the isolation of a panel of Rituximab-specific phage clones. The alignment of their insert peptide sequence resulted in the definition of two distinct sets of peptides, each expressing a specific motif. The first set of peptides expressed the motif (a/sNPS), matching the human CD20 ^170^(ANPS)^173^, and the second set consisted of peptides all expressing the motif WPxWLE, lacking homology to CD20, and thus likely mimicking a conformational or discontinuous Rituximab-specific epitope [15,16,17,18]. The motifWPxWLE also matched the reverse-oriented motif, ELWxPW, expressed by the acid sphingomyelinase-like phosphodiesterase 3b precursor (ASMLPD) [17], suggesting a possible cross-reactivity of Rituximab with this enzyme [17,19].

Sera antibodies elicited with WPxWLE motif-expressing peptides strongly reacted with the immunogen peptide, but displayed low-affinity binding to CD20^+^ B lymphoid cells. The reason for the lower reactivity was possibly the recognition by anti-peptide sera of motif amino acids different from the WPxWLE motif recognized by Rituximab [20,21].

Therefore, in the present investigation we conducted a more detailed epitope-specificity analysis using a panel of mAb against the Rituximab-specific peptide Rp5-L, one of the phage clone-derived insert peptides, containing the minimal epitope required for Rituximab binding [18]. We found that even with a 13-mershort peptide, the motif recognized by mAb elicited with Rp5-L (immunogenic motif) was different from the (Rituximab-specific) “WPxWLE” motif (antigenic motif). These findings may lie at the basis of epitope spreading and should be considered when designing peptide-based vaccines.

## 2. Results

**Figure 1:**
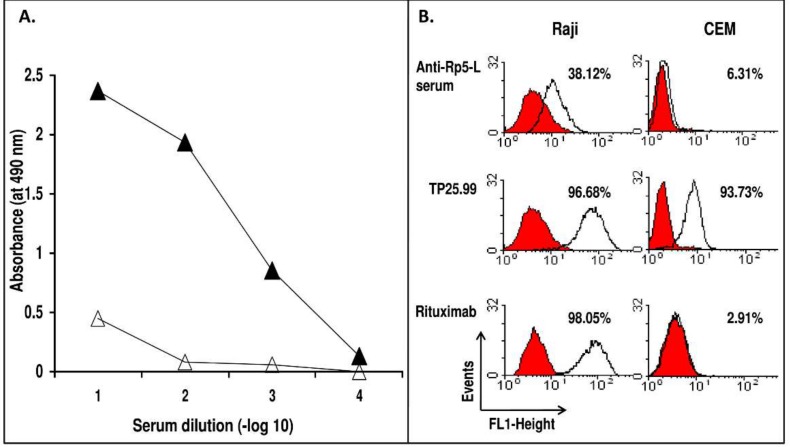
(**A**) Specificity of the reactivity of sera antibodies elicited in a BALB/c mouse immunized with Rituximab-specific peptide Rp5-L. 10-fold serial dilutions of serum were added to wells previously coated with Rp5-L peptide (closed triangle). Following a 4h incubation at R/T, bound antibodies were detected with horseradish peroxidase (HRP)-conjugated goat anti-mouse IgG. The Qp-1a peptide was used as a negative control (open triangle). (**B**) The occurrence of anti-CD20 Ab in serum from mice immunized with Rp5-L. Anti-Rp5-L serum (diluted 1:20) was added to rabbit IgG-treated CD20^+^ B lymphoid Raji cells. Bound antibodies were detected with an appropriate dilution of fluorescein isothiocyanate (FITC)-conjugated goat anti-mouse IgG. Rituximab, the anti-HLA class I mAb TP25.99 and the CD20^−^ human T lymphoid CEM cells were used as controls.

Immunization of a BALB/c mouse with Rp5-L elicited moderate titers of specific anti-peptide Ab (Figure 1A). Sera drawn on day 35, which displayed the highest binding with the immunogen, specifically reacted with CD20^+^ human B lymphoid Raji cells (38.12% stained cells), even if to a lesser extent than Rituximab (98.05% stained cells). The specificity was indicated by the lack of reactivity with CD20^−^ human T lymphoid CEM cells, and by the binding of the mouse monoclonal anti-HLA Class I mAb TP25.99 to both cell lines (Figure 1B).

**Figure 2:**
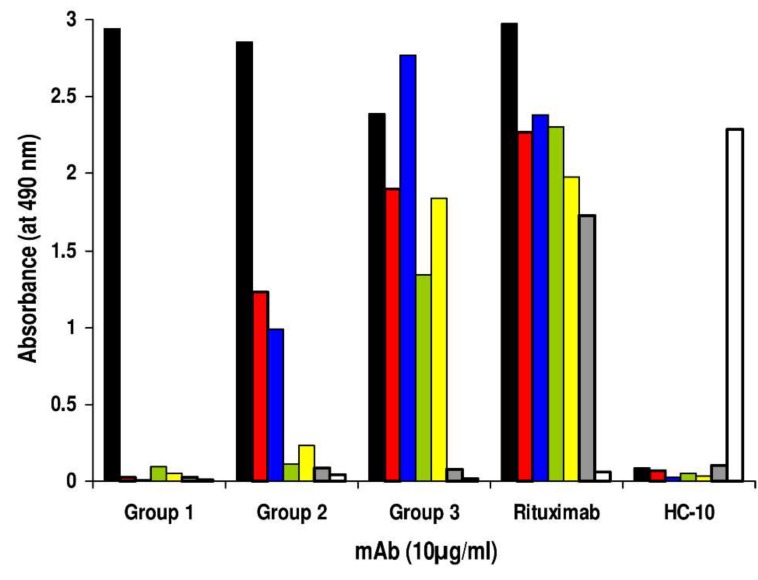
Binding assay to define the differential reactivity of anti-Rp5-L mAb with a panel of Rituximab-specific peptides. Hybridoma-supernatants secreting anti-Rp5-L mAb were added to wells previously coated with Rituximab-specific peptides Rp5-L (black bar), Rp1-L (red bar), Rp10-L (blue bar), pASMLPD (green bar), Rev-pASMLPD (yellow bar), RpCD20-L (grey bar), and Qp-1a (white bar) for 4 hours at R/T. Peptide-antibody binding was detected with HRP-conjugated goat anti-mouse Ig. Rituximab, the anti-HLA class I mAb HC-10, and the peptide Qp-1a were used as controls. The data are representative of two experiments.

The hybridization of splenocytes from this mouse with mouse myeloma cells P3-X63-Ag8.653 yielded 780 hybridomas. Testing of their spent medium with Rp5-L in binding assay detected activity in 59 supernatants. Anti-Rp5-L mAb supernatants were then tested for their reactivity with the panel of Rituximab-specific peptides bearing the WPxWLE motif (Table 1). Three groups of mAb were identified according to their immunoreactivity profiles (Figure 2). The first group (Group 1; #21 hybridomas supernatants; 35.5%) included mAb reacting only with Rp5-L. The second group (Group 2; #26 hybridomas supernatants; 44.06%) included anti-Rp5-L mAb cross-reacting with Rp1-L and Rp10-L. Finally, the third group (Group 3; #12 hybridomas supernatants; 20.33%) included mAb reacting with all linear Rituximab-specific peptides, but not with RpCD20-L. The binding was specific since neither mAb reacted with the unrelated peptide Qp-1a, nor did Qp-1a-specific mAb HC-10 react with Rituximab-specific peptides (Figure 2). In further investigations, mAb belonging to Group 1 were excluded because they recognized Rp5-L, but none of the other Rituximab-specific peptides. mAb FE-718 and FE-341, which showed the highest reactivity with Rp5-L, besides reacting similarly to Rituximab with Rp1-L and Rp10-L, were selected for further study as representative of Group 2 and Group 3, respectively.

**Figure 3:**
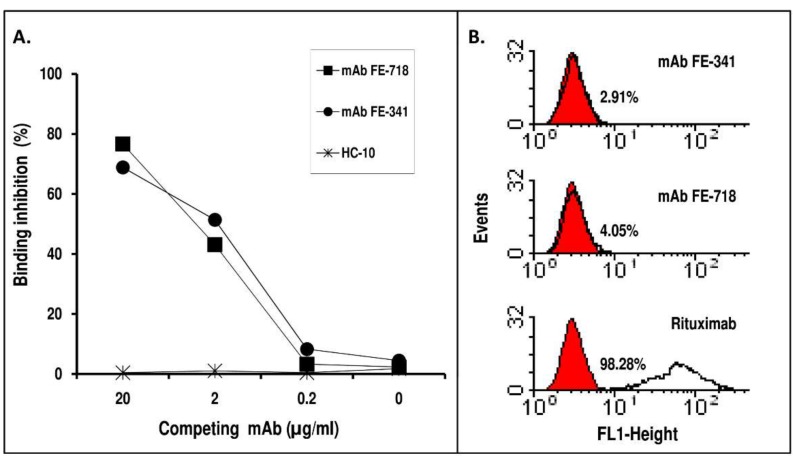
(**A**) Competitive binding assay to define the relative avidity of mAb FE-718 and FE-341 for Rp5-L. Ten-fold serial dilution of mAb FE-718 and FE-341 were mixed with an equal volume of an appropriate dilution of Rituximab and added to wells previously coated with Rp5-L. Following a 2-hour incubation at R/T, Rituximab-bound was detected with HRP-conjugated goat anti-human Ig (Fc portion). The anti-HLA class I mAb HC-10 was used as control. Results are expressed as percentage inhibition of binding compared with binding in the absence of inhibitor. The data are representative of two experiments. The O.D. of uninhibited Rituximab was 2.41±0.23 S.D. (**B**) Reactivity of mAb FE-718 and FE-341 with CD20^+^ B lymphoid cells. mAb FE-718 and FE-341 were added to rabbit IgG-treated CD20^+^ B lymphoid Raji cells. Bound antibodies were detected with an appropriate dilution of FITC-conjugated goat anti-mouse IgG. Rituximab was used as control.

These mAb were tested in a competitive binding assay to evaluate their ability to inhibit the binding of Rituximab to Rp5-L. As shown in Figure 3A, both mAb dose-dependently, and to a similar extent, inhibited Rituximab binding to Rp5-L. The inhibition was specific in that the unrelated anti-HLA class I HC-10 did not affect the binding. The data indicate that the estimated binding avidity of mAb FE-781 and FE-341 for Rp5-L was similar to that of Rituximab, because 2 μg/mL of Rituximab binding to Rp5-L was inhibited by ~50% by 2 μg/mL of mAb FE-718 or FE-341.

mAb FE-718 and FE-341 were then tested by flow cytometry to assess their ability to bind CD20^+^ human B lymphoid cells. Neither mAb FE-718 nor mAb FE-341 recognized the cell surface CD20 (Figure 3B). These results indicate that mAb FE-718 and FE-341 display unique specificities, even if they recognize the same peptides seen by Rituximab.

**Figure 4:**
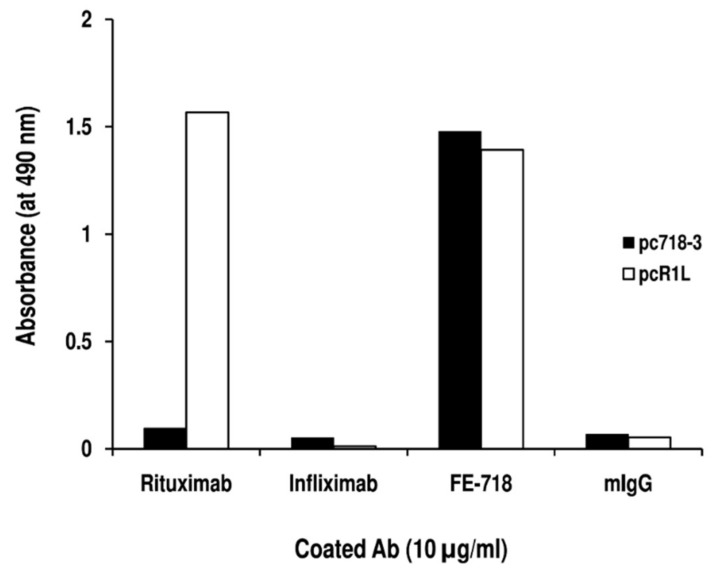
Reactivity of Rituximab with the mAb FE-341-specific phage clone pc718-3. Supernatants from pc718-3 and pcR1L phage clones were diluted 16-fold and added to wells previously coated with mAb or mouse IgG (mIgG). After a 4h incubation, the antibody-phage clone interaction was detected with HRP-conjugated anti-M13 mAb. The data are representative of two experiments.

To define the immunogenic motifs recognized by mAb FE-718 and FE-341, these mAb were used to pan the 7-mer and 12-mer PDPL, respectively. After panning with mAb FE-718, 50 colonies were randomly selected, and immunoscreening showed that 18 clones (36%) reacted specifically with mAb FE-718. The binding was specific, since no reactivity with mouse IgG was detected (Table 2). Nucleotide sequencing of all 18 phage clone inserts identified 3 distinct sequences (pc718-1, pc718-2, and pc718-3) (Table 2). After panning with mAb FE-341, 70 colonies were randomly selected and ELISA screening showed that 39 clones (55.7%) reacted specifically with mAb FE-341. Nucleotide sequencing of 18 of these phage clone inserts identified 5 distinct sequences (pc341-1, pc341-2, pc341-3, pc341-4 and pc341-5) (Table 2). Alignment of the 3 sequences from mAb FE-718-specific phage clones and of the 5 sequences from mAb FE-341-specific phage clones identified the WPxxL motif (Table 2). Comparison of the mAb FE-718 and FE-341 -specific motif, “^1^WPxxL^5^”, with the one recognized by Rituximab, “^1^WPxWLE^6^”, showed that both W^4^ and E^6^ could be critical for Rituximab binding. To test this hypothesis, we evaluated the reactivity of Rituximab with the mAb FE-718 specific phage clone (pc718-3) bearing the “MWPKWLP” insert. As shown in Figure 4, Rituximab did not bind pc718-3. The specificity of the assay was confirmed by the reactivity of Rituximab with its specific-phage clone R1L (pcR1L), bearing the “WPRWLEN” insert. Instead, mAb FE-718 reacted with both pc718-3 and pcR1L phage clones. These results demonstrated that only E^6^ is essential for Rituximab binding. 

## 3. Discussion

Immunization with peptides mimicking the binding site of therapeutic antibodies hasshown encouraging results in preclinical studies, eliciting antibodies against the nominal antigen [22,23,24].

Here we showed that immunization of a BALB/c mouse with the 13-mer linear peptide Rp5-L, bearing the “dominant” antigenic motif <^1^WPxWLE^6^> (Rituximab-specific), elicited a panel of mAb that recognized the immunogenic <WPxxL> motif, accounting for only a portion of the original antigenic <WPxWLE> motif. In fact, “W” at position 4 and “E” at position 6 were no longer required for peptide recognition by mAb FE-718 and FE-341, thus making their antigen recognition less restricted, and expanded to a wider array of potential antigens that are not necessarily linked to the CD20 system. 

In addition, the role of amino acid side chains and the Rp5-L backbone in the reactivity with FE-718 and FE-341 could not be investigated as we did for Rituximab and Rp5-L [18], since the crystallographic structures of mAb FE-718 and FE-341 are not yet available. Although the antigenic specificity of mAb FE-718 and FE-341 is only a portion of the whole antigenic-specificity-repertoire recognized by anti-Rp5-L antibodies, the results may point to the molecular basis underlying the failure of Rp5-L to consistently induce high titers of anti-CD20 antibodies [17]. They also suggest that even with a short peptide (much shorter than the peptide corresponding to the whole extracellular portion of CD20 [14]), a skewed immune response can be generated, that may explain the molecular basis of epitope spreading.

Epitope spreading, namely the development of an immune response to epitopes different from, and non-cross-reactive with, the dominant antigenic determinant on a vaccine peptide [25,26,27,28], has been previously reported. For instance, in patients immunized with HER/neu-mimicking peptide, the immune response elicited with HER/neu was also effective against p53, an intracellular protein structurally unrelated to HER/neu [26]. The phenomenon of epitope spreading has so far been attributed to a two-step process. In the first step, target cells are lysed by the vaccine peptide-induced immune response and intracellular self-antigens are released; in the second step, the released self-highly immunogenic antigens can, in turn, prime the immune system to generate a novel immune response obviously unrelated to the previous one [29]. The recognition by mAb raised with the Rituximab-specific peptide Rp5-L of a different antigenic motif from the one recognized by Rituximab, indicates that these mAb might react with proteins belonging to other antigen systems, suggesting an additional, intrinsic mechanism for epitope spreading, regardless of whether the release of intracellular antigen occurred. If this conclusion is correct, then epitope spreading may constitute an intrinsic property of the immune response.

Epitope spreading generated by this mechanism can justify why immunization with mimotope peptides failed to elicit antibodies against the naïve proteins in different antigen systems in cancer [30] and in infectious diseases [31]. Indeed, peptides mimicking high molecular weight-melanoma-associated antigen (HMW-MAA)-associated epitope recognized by the anti-HMW-MAA mAb VT80.12 and VF1-TP43 elicited anti-peptide antibodies, but failed to induce anti-HMW-MAA antibodies in rabbits [30]. Similarly, peptides mimicking the capsule component glucuronoxylomannan (GXM) of *Cryptococcus neoformans* did not elicit anti-GXM antibodies in mice, despite the induction of high titers of anti-peptide antibodies [31]. In the same way, as in our system, Luo et al. found that immunization with a peptide mimicking the epitope of the anti-HMW-MAA mAb GH786 induced an antigen-specific immune response but at lower affinity compared to anti-peptide antibodies [32]. However, in none of these studies were the molecular bases of these findings investigated.

If the difference between the antigenic and the immunogenic motif [33,34,35] lies at the basis of the skewed immune response, epitope spreading, and mimotope failure to elicit a strong nominal antigen-specific response [31,32,36], then one possible strategy to overcome this limitation would be to appropriately substitute the amino acids surrounding the antigenic motif in order to narrow the focus of the immune response onto the desired epitope. Unfortunately, there are no molecular rules to follow for this substitution in order to ensure that the antigenic motif coincides with the immunogenic motif. Site-directed mutagenesis could be a way to optimize the mimotope immunogenicity [37,38,39]. Alternatively, the availability of a panel of peptides bearing the same antigenic motif, but with different strings of amino acids surrounding the antigenic motif, could be a way to narrow the focus of the immune response more strictly onto the antigenic motif. The feasibility and effectiveness of this strategy has been supported by our recent findings showing that vaccination with a mixture of small cyclic peptides, all expressing the same antigenic motif recognized by Rituximab on CD20, but with different amino acid sequences outside the motif, induced B cell depletion and prolonged survival in (New Zealand black/New Zealand White) F1 systemic lupus erythematosus -prone treated mice [21], the noteworthy biological effects observed with the passive administration of Rituximab. Nevertheless, further studies are warranted to identify an effective strategy for peptide design in vaccine-immunotherapy.

## 4. Materials and Methods

### 4.1. Animals 

The experimental procedures were approved by the Animal Ethics Committee of the University of Bari Medical School (D.M. n. 9072013-B). Female BALB/c mice, 8–12 weeks old, were purchased from Charles River Breeding Laboratories (Milan, Italy). 

### 4.2. Cells 

The human B-lymphoid cell line Raji, the T-lymphoid cell line CEM, and the mouse myeloma cell line P3-X63-Ag8.663 were grown in RPMI 1640 medium supplemented with 10% FCS and 5 mM l-glutamine.

### 4.3. Conventional Reagents, mAb, Peptides and PDPL

Unless otherwise specified, all chemicals were purchased from Sigma-Aldrich (St. Louis, MO, USA). The anti-CD20 mAb Rituximab and the anti-TNFα mAb Infliximab were purchased from IDEC Pharmaceutical Corporation (San Diego, CA, USA). The anti-HLA class I mAb HC-10 and TP25.99 had been previously characterized [40]. mAb were purified from the ascitic fluid by sequential precipitation with caprylic acid and ammonium sulfate [41]. Horseradish-peroxidase (HRP)- or fluorescein isothiocyanate (FITC)-conjugated goat antibodies to the Fc portion of human or mouse IgG were purchased from Jackson Immunoresearch Laboratories (Avondale, PA, USA). HRP-anti-M13 mAb was purchased from GE Healthcare Life Sciences (Milan, Italy). Rituximab-specific peptides Rp5-L, Rp1-L, Rp10-L, Rev-pASMLPD, pASMLPD and RpCD20-L had been previously characterized [16,17]. HC-10-specific peptide Qp-1a has already been described [40]. The 7- and 12-mer PDPLs were purchased from New England Biolabs (Beverly, MA, USA). The Rituximab-specific phage clone pcR1L had been previously characterized [17].

### 4.4. Preparation of Anti-Rp5-L mAb

Rp5-L was coupled to keyhole limpet hemocyanin (KLH) as previously described [18]. A BALB/c mouse was immunized by an intraperitoneal injection of 10 μg of KLH-Rp5-L, and mixed with CFA (Thermo Fisher Scientific, Waltham, MA USA). The mouse treatment was then boosted with 10 μg of the same immunogen in IFA on days 7, 14, and 21. Serum samples were harvested on days 28, 35, 42 and 94. On day 101 the mouse was sacrificed, the spleen was removed and splenocytes were fused with mouse myeloma cells P3-X63-Ag8.653 according to standard procedures. FE-718 and FE-341 hybridomas were subcloned for two rounds using the limiting dilution method.

### 4.5. Binding Assay

The binding assay to test the reactivity of immune sera or mAb with Rp5-L was performed in 96-well polyvinyl-chloride microtiter wells as previously described [17], with minor modifications. Briefly, plates were coated with a PBS solution containing 10μg/mL BSA peptide. After washing, 50 μL of a 10-fold dilution of serum samples, or supernatant containing mAb or purified mAb, were added to each well. Following a 4h incubation at R/T, plates were washed and incubated with an appropriate dilution of peroxidase conjugated xeno-antisera to mouse or human IgG (Fc portion) for 1 h at R/T. Next, plates were washed and developed by the addition of the *o*-phenylenediamine (OPD) substrate solution. 

In the competitive binding assay, wells were coated with 10 µg/ml BSA Rp5-L o/n at 4 °C. After one washing and blockade of free-protein binding sites, wells were incubated with a subsaturating concentration of Rituximab (2 μg/mL) mixed with an equal volume of PBS containing competing mAb in 10-fold serial dilutions, starting at 20 µg/mL, for 2 h at R/T. The wells were then washed and the Rituximab-peptide interaction was detected with an appropriate dilution of HRP-conjugated goat anti-human IgG.

### 4.6. Flow Cytometry

The reactivity of anti-Rp5-L serum or mAb with CD20^+^ B lymphoid Raji cells was performed as previously described [17], with minor modifications. Briefly, 1:20 dilutions of anti-peptide mouse antisera or 5 μg/mL of mAb were mixed with 5 × 10^5^ cells previously preincubated with 50 μg/mL of rabbit IgG. After a 30min incubation on ice, cells were washed and incubated with an appropriate dilution of FITC-conjugated antibodies to mouse or human IgG, and immunofluorescence was measured using a FACScan cytometer.

### 4.7. Biopanning, Immunoscreening, and Sequence Analysis

The panning of the peptide library with mAb FE-718 or FE-341 was performed as previously described [42], the only difference being that the mouse IgG were used to remove isotype- and allotype-specific phage particles. Phage clones were tested for specificity to mAb FE-718 or FE-341 in an indirect ELISA, using mouse IgG as a negative control, as previously described [16]. mAb-specific phage clone inserts were sequenced at the Eurofins Genomics sequencing facility (Ebersberg, Germany). Nucleotide sequence analysis was performed as previously described [18].

## Figures and Tables

**Table 1:** Peptides used in the immunochemical assay.

Sequence Origin	Peptide	
	Denomination	Sequence
**Rituximab-specific peptides**		
	Rp5-L	QDKLTQ**WPKWLE**g
	Rp1-L	**WP**R**WLE**N
	Rp10-L	ITP**WP**H**WLE**RSSg
**ASMLPD**		
	Rev-pASMLPD	^163^SL**WP**K**WLE**AIQ^153^
	pASMLPD	^153^QIA**ELW**K**PW**LS^163^
**Human CD20**		
	RpCD20-L	^165^YNCEPANPSEKNSPSTQYCY^184^

Motif amino acids are in bold type. ASMLPD, acid sphingomyelinase-like phosphodiesterase 3b precursor. “g” is a glycine residue of the phage which flanks the C-terminus of the peptide insert.

**Table 2:** Definition of mAb FE-341- and FE-718-specific motifs.

Phage Clone Insert #	Clones, *n* (%)	Deduced Amino Acid Insert Sequence ^(a)^	Specificity of Reactivity (A_490nm_)	
			mAb	mIgG
pc718-1	5 (27.7%)	**WP**HV**L**PE	1.754 ± 0.01	0.123 ± 0.002
pc718-2	2 (11.1%)	K**WP**QY**L**S	1.833 ± 0.14	0.144 ± 0.07
pc718-3	11 (61.1%)	M**WP**KW**L**P	1.92 ± 0.044	0.107 ± 0.02
FE-718 motif		**WP**—-**L**		
pc341-1	4 (22.2%)	SLKMPH**WP**HL**L**P	1.644 ± 0.01	0.167 ± 0.002
pc341-2	1 (5.5%)	QHVNLAR**WP**WQ**L**	1.834 ± 0.021	0.111 ± 0.013
pc341-3	10 (55.5%)	TQLG**WP**HSIGDA	1.421 ± 0.08	0.172 ± 0.1
pc341-4	2 (11.1%)	HSS**WP**RH**L**DPPQ	1.962 ± 0.013	0.069 ± 0.002
pc341-5	1 (5.5%)	Q**WP**NE**L**RNSGLS	1.718 ± 0.032	0.098 ± 0.011
FE-341 motif		**WP**—-**l**		
Rituximab motif		**WP**-**WLE**		

^(a)^ Multiple alignment was performed with Clustal Omega at EMBL-EBI (https://www.ebi.ac.uk/Tools/msa/clustalo/). Amino acids matching those of the motif recognized by Rituximab are in bold type.
